# “Special K” Drug on Adolescent Rats: Oxidative Damage and Neurobehavioral Impairments

**DOI:** 10.1155/2019/5452727

**Published:** 2019-03-14

**Authors:** Sabrina de Carvalho Cartágenes, Luanna Melo Pereira Fernandes, Taiana Cristina Vilhena Sarmento Carvalheiro, Thais Miranda de Sousa, Antônio Rafael Quadros Gomes, Marta Chagas Monteiro, Ricardo Sousa de Oliveira Paraense, Maria Elena Crespo-López, Rafael Rodrigues Lima, Enéas Andrade Fontes-Júnior, Rui Daniel Prediger, Cristiane Socorro Ferraz Maia

**Affiliations:** ^1^Laboratory of Pharmacology of Inflammation and Behavior, Pharmacy Faculty, Institute of Health Sciences, Federal University of Pará, Belém, Pará, Brazil; ^2^Laboratory of Microbiology and Immunology of Teaching and Research, Pharmacy Faculty, Institute of Health Science, Federal University of Pará, Belém, Pará, Brazil; ^3^Laboratory of Molecular Pharmacology, Institute of Biological Sciences, Federal University of Pará, Belém, Pará, Brazil; ^4^Laboratory of Functional and Structural Biology, Institute of Biological Sciences, Federal University of Pará, Belém, Pará, Brazil; ^5^Department of Pharmacology, Center of Biological Sciences, Federal University of Santa Catarina, Florianópolis, Santa Catarina, Brazil

## Abstract

Ketamine is used in clinical practice as an anesthetic that pharmacologically modulates neurotransmission in postsynaptic receptors, such as NMDA receptors. However, widespread recreational use of ketamine in “party drug” worldwide since the 1990s quickly spread to the Asian orient region. Thus, this study aimed at investigating the behavioral and oxidative effects after immediate withdrawal of intermittent administration of ketamine in adolescent female rats. For this, twenty female Wistar rats were randomly divided into two groups: control and ketamine group (*n* = 10/group). Animals received ketamine (10 mg/kg/day) or saline intraperitoneally for three consecutive days. Three hours after the last administration, animals were submitted to open field, elevated plus-maze, forced swim tests, and inhibitory avoidance paradigm. Twenty-four hours after behavioral tests, the blood and hippocampus were collected for the biochemical analyses. Superoxide dismutase, catalase, nitrite, and lipid peroxidation (LPO) were measured in the blood samples. Nitrite and LPO were measured in the hippocampus. The present findings demonstrate that the early hours of ketamine withdrawal induced oxidative biochemistry unbalance in the blood samples, with elevated levels of nitrite and LPO. In addition, we showed for the first time that ketamine withdrawal induced depressive- and anxiety-like profile, as well as short-term memory impairment in adolescent rodents. The neurobehavioral deficits were accompanied by the hippocampal nitrite and LPO-elevated levels.

## 1. Introduction

Adolescence consists of the transition from childhood to adulthood [1]. The age group from 10 to 19 years old comprises the adolescence period in humans [2], which corresponds to 28 to 42 days postnatal in rodents [3]. During this period, individuals suffer physical, psychosocial, and cognitive transformations related to adolescent brain maturation [4]. These changes provide a biological basis for adolescent-specific behaviors during this period with repercussions in adult life [1].

Actually, important structural and functional changes in synaptic plasticity and neural connectivity occur during the development and maturation of the Central Nervous System (CNS). The brain refines its connections, generating greater communication between the CNS regions, enabling better interaction and increased complexity in specific functions [5]. Thus, the adolescent brain is vulnerable to the harmful effects of drug abuse [1, 6]. Therefore, exposure to psychotropic drugs during the critical stages of development in adolescents may disturb the processes of maturation and plasticity, leading to behavioral and cognitive deficits [5].

Among adolescents, the use of prevalent substance abuse known as “club drugs” (drugs at nightclubs, raves, and bars) occurs. These substances have several psychotropic effects and are associated with a variety of levels of neurotoxicity, dependence, and adverse effects. Among these drugs, ketamine can be highlighted [7].

Ketamine is a neuroleptic anesthetic agent in clinical use since the 1960s. Chemically, it presents itself as a [2-O-chlorophenyl-2-(methylamino) cyclohexanone] [8], consisting of a phencyclidine derivative. When introduced in clinical practice, it was regarded as an ideal and complete anesthetic drug, since it provides all the required components of surgical anesthesia (i.e., pain relief, immobility, amnesia, and loss of consciousness) [9]. Pharmacologically, ketamine modulates neurotransmission at postsynaptic receptors such as N-methyl-D-aspartate (NMDA) glutamate receptors and gamma-aminobutyric acid (GABA) receptors. As an uncompetitive antagonist, ketamine blocks NMDA receptor and induces a dissociative anesthesia [10].

Recreational use of ketamine was first reported in 1967 among subjects with access to the drug, particularly medical professionals [11]. The ketamine misuse spread beyond this group to the community-at-large, firstly in the United States followed by other countries in the world, associated with the “rave” dance subculture of the 1980-1990s. On the streets, ketamine is known as “angel dust,” Special K, Cat Valium, “K,” or “Kit Kat” [11, 12].

Low doses of ketamine induces distortion of time and space, hallucinations, and mild dissociative effects. At large doses, ketamine induces a more severe dissociation commonly referred to as a “K-hole,” wherein the user experiences intense detachment to the point that their perceptions appear completely divorced from their previous reality [13]. In rodents, acute subanesthetic doses of ketamine produce a schizophrenia-like symptomatology, including hyperlocomotion, enhanced stereotyped behaviors, cognitive and sensorimotor gating deficits, and impaired social interactions [14–16].

It is important to emphasize that drug abuse generates toxic effects in different organs, which depends on the administration pathway used by the addict [17, 18]. The affected organs by drug addiction consists of the brain, heart, liver, and kidneys, generally through oxidative stress processes that may derive from direct or indirect effects of the chemical compound, as well as during the drug withdrawal [18]. In fact, studies in humans have reported that ketamine induces neurotoxicity through oxidative stress mechanisms [13]. In rodents, ketamine causes a compensatory overexpression of NMDA receptors and increased the Ca^2+^ levels [19], resulting in Ca^2+^ accumulation that leads to mitochondrial excitotoxic injury and production of reactive oxygen species (ROS) [19, 20].

The neurobehavioral alterations generated by the recreational use of ketamine indicate gender differences in the substance-related epidemiology, social factors, biological responses, and progression to dependence [21, 22]. For example, the consumption of some licit substances, such as alcohol, in women progresses faster than in men. Besides, women present more serious problems and greater health-related consequences with the use of licit and illicit substances, due to the dopaminergic system in the limbic regions that, in part, mediate the sexual difference in drug abuse [21, 23]. Additionally, the stimulus and relapse by the search for drugs in abstinent individuals is different between men and women, in which women are more likely to initiate misuse and relapse after withdrawal than men [21, 24].

In fact, studies exploring different human responses of sex to ketamine are scarce [21]. An animal study designed by Winters et al. [25], which compared the different analgesic effects of ketamine in male and female rats, indicated that females were much more sensitive to the drug effects than males [21, 26, 27]. Moreover, females are more sensitive to neurotoxicity of ketamine and cerebral neural loss than males [28] and are more susceptible to ketamine withdrawal symptoms and adverse effects, especially in cognitive deficits [21, 29].

Thus, in the present study, we investigated the oxidative effects and neurobehavioral alterations after intermittent administration of ketamine in adolescent female rats.

## 2. Materials and Methods

### 2.1. Animals and Ethical Aspects

Twenty female Wistar rats (25 days old, weight 60-100 g) were provided from the Federal University of Pará (UFPA) and maintained in collective cages (five animals per cage). Animals were housed (12 h light/dark cycle, lights on 7:00 a.m.) with food and water *ad libitum*. This study followed the *NIH Guide for the Care and Use of Laboratory Animals*, and it was approved by the Committee for Ethics in Experimental Research with Animals of the Federal University of Pará (license number BIO 224-14).

### 2.2. Study Design

Animals were randomly divided into two groups (10 animals/group). At 35^th^ days old, animals received saline (control) or ketamine 10 mg/kg/day intraperitoneally (i.p) [30] for three consecutive days. Rats in the ketamine group presented with cataleptic immobility within 1 min after administration of 10 mg/kg ketamine i.p. Dextroketamine hydrochloride (Cristália, Brazil) was diluted in saline prior to administration at a volume/body weight ratio of 0.1 mL/100 g [31].

Gass et al. [32] used ketamine (5, 10 e 25 mg/kg) to investigate the modulatory effect of acute ketamine administration on functional connectivity in the hippocampus and prefrontal cortex system of the rat and proved that ketamine produced a dose-dependent increase in the functional connectivity. Based on these references and our preliminary experiments, we selected the dosages of ketamine 10 mg/kg/day. Besides, to mimic the pattern of social behavior among adolescents, we performed the 3 ON-4 OFF paradigm that has been validated by our group on the study related to drug addiction during adolescence.

### 2.3. Behavioral Assays

Animals were acclimated for 1 h in the test room with attenuation of noise levels and low illumination (12 lux). To obtain the behavioral effects of ketamine on the immediate withdrawal, three hours after the last administration of saline or ketamine (37th days old), the animals were submitted to the open field (OF), elevated plus-maze (EPM), forced swimming (FS), and step-down inhibitory avoidance task. All behavioral assays were videotaped and analyzed by ANY-maze software (San Diego, CA), except for the rearing parameter of open field (OF) that was used as a manual counter and inhibitory avoidance task. In addition, blindness of the study was preserved.

#### 2.3.1. Open-Field Test

In the OF test, animals were individually placed in the center of a black square arena (100 × 100 × 40 cm) and were permitted with spontaneous ambulation for five minutes. To evaluate the horizontal and vertical locomotor activity, the total distance traveled (in meters) and number of rearing were measured [33].

#### 2.3.2. Elevated Plus-Maze (EPM)

After OF test, animals were submitted to EPM. The maze consists of two open (no walls, 50 × 10 × 1 cm) and two enclosed (50 × 10 × 40 cm) arms, arranged perpendicularly, and elevated 50 cm above the floor. Each animal was placed on the center of the equipment, facing the closed arm. Animals were able to explore the apparatus for 5 minutes. The parameters measured were the percentage of open arm entries (% OAE), the percentage of open arm time (% OAT) [34], and the frequency of enclosed arm entries (EAE) [35]. The % OAE and % OAT were calculated according to the formula [(open/total) × 100]. Entry into an arm was defined when the animal places all four paws onto the arm.

#### 2.3.3. Forced Swimming (FS) Test

After EPM, animals were gently placed individually in a vertical Plexiglas cylinder (high 50 cm by 30 cm diameter) filled with 40 cm water column at a temperature of 23 ± 1°C. The test lasted for 5 min, which the first two minutes consisted of habituation of animals, and the last three minutes measured the immobility time, according to the criteria of Porsolt [36]. Immobility time (i.e., animal floated passively, with only small movements to keep the nose above the surface) and climbs (upward-directed movements) were measured during the three final minutes of the test.

#### 2.3.4. Inhibitory Avoidance Task

After FS test, animals were conducted to the habituation session (180 s) of inhibitory avoidance test. The apparatus consists of an acrylic box (50 cm × 25 cm × 25 cm), with a floor comprising parallel copper bars (1 mm diameter) connected to an electrical stimulator and a secure platform (7 cm wide × 2.5 cm high). After 24 hours (training session), animals were resubmitted to the secure platform of the apparatus, and immediately after stepping down on the grid, they received a 0.4 mA, 1.0 s scrambled foot shock. During test sessions (1.5 h after training session), animals were placed on the secure platform and the step-down latency (maximum 180 s) was used as measure of retention (short-term memory) [37].

### 2.4. Oxidative Biochemistry Assays

To avoid interference of behavioral assays on oxidative stress analysis [38], 24 h after the last behavioral task, animals were sacrificed by cervical dislocation for collection of blood contents by intraventricular cardiac puncture preceded by thoracotomy. Concomitantly, the brain was removed from the cranial box and washed with saline at 4°C. Then, the hippocampus was dissected, frozen in liquid nitrogen, and stored in a freezer -80°C until analysis. For analysis, the samples were thawed and resuspended in 20 mM Tris-hydrochloride (Tris-HCl) buffer, pH 7.4, at 4°C for sonic disintegration. The results were expressed as percentages of the control groups.

#### 2.4.1. Oxidative Biochemistry in the Blood

1. Determination of Serum Malondialdehyde (MDA). Determination of malondialdehyde (MDA) is a method that evaluates the lipid peroxidation and acts as an indicator of cell damage. The technical procedure was performed according to the protocol proposed by Kohn and Liversedge [39], adapted by Percario et al. [40]. The method is based on the reaction of MDA, among other substances, with thiobarbituric acid (TBA 10 nM; Sigma-Aldrich T5500), in pH 2.5 and high temperature (94°C × 60 min). The formation of the complex MDA-TBA generates a pink color that was measured by the spectrophotometric method (535 nm) and concentrations expressed in nmol/mL. 1,1,3,3-Tetraethoxypropane (Sigma-Aldrich; T9889) was used for the implementation of the standard curve.
2. Determination of Serum Nitric Oxide (NO). The determination of nitric oxide (NO) was based according to the method described by Granger et al. [41]. In this assay, nitrate (NO_3_-) present in the serum samples was reduced to nitrite using nitrate reductase, and the nitrite concentration was determined by the Griess method. Briefly, 100 *μ*L of the serum supernatant was incubated with an equal volume of Griess reagent for 10 minutes at room temperature. The absorbance was then measured on a plate scanner (SpectraMax 250, Molecular Devices, Menlo Park, CA, USA) at 550 nm. Nitrite (NO_2_-) was determined using a standard curve generated using sodium nitrate (NaNO_2_). Nitrite production was expressed in *μ*M.
3. Measurement of Catalase (CAT) Activity. Catalase (CAT) activity was determined following the method described by Bleuter [42], measuring the rate of enzymatic decomposition of hydrogen peroxide (H_2_O_2_) (10 mM) to H_2_O and O_2_. The decay of H_2_O_2_ was measured using ultraviolet spectrophotometry at 240 nm, and enzyme activity was expressed in CAT units, where one unit is the amount of enzyme needed to hydrolyze one *μ*mol of H_2_O_2_/min/mg protein.
4. Measurement of Superoxide Dismutase (SOD) Activity. Superoxide dismutase (SOD) activity was performed according to the procedure recommended by McCord and Fridovich [43]. This method evaluated the ability of SOD to catalyze the conversion of O_2_- to H_2_O_2_ and O_2_. SOD activity was measured using ultraviolet (UV) spectrophotometry at a wavelength of 550 nm and was expressed in nmol/mL.

#### 2.4.2. Oxidative Biochemistry in the Tissue Samples

1. Concentration of the Hippocampal Nitrite Levels. The nitrite levels were determined based on a reaction with Griess reagent (0.1% naphthylethylenediamine and 1% sulfanilamide in 5% phosphoric acid; 1 : 1). An aliquot of tissue homogenate was centrifuged at 21,000 g for 10 min at 4°C, and supernatant was used to analyze the nitrite levels. Fifty microliters of supernatant or standard sodium nitrite solution was added to 50 microliters of Griess reagent and incubated for 20 minutes at room temperature. The absorbance was measured at 550 nm wavelength and compared with the standard curve.
2. The Hippocampal Lipid Peroxidation Levels. Lipid peroxidation was determined based on the measurement of the MDA and 4-hydroxyalkenals (4-HA) levels. An aliquot of homogenate was centrifuged at 2,500 g for 30 min at 4°C, and supernatant was used for reaction with N-methyl-phenyl indole (NMFI) and methanesulfonic acid at 45°C, during 40 min, yielding a stable chromophore measured at 570 nm wavelength and compared with the standard curve of MDA.
3. Determination of Protein Content. Total protein content in the supernatants was assayed using the Bradford [44] methodology. An aliquot of homogenate was incubated with Bradford reagent (5% ethanol; 8.5% phosphoric acid; 0.25% Coomassie Brilliant Blue G-250) for 5 min at room temperature. The absorbance was measured at 570 nm and compared to standard solutions of bovine serum albumin. The results were used for the correction of the MDA and nitrite levels.

### 2.5. Statistical Analysis

The data on the inhibitory avoidance task are shown as median (interquartile range) of step-down latencies. Comparisons of both training and test session step-down latencies between the groups were performed with Mann-Whitney. The remaining data are expressed as mean ± standard error of the mean (SEM) (*n* = 10 animals per group for behavioral assays and *n* = 5 animals per group for oxidative stress parameters). Statistical comparisons between the groups were performed using Student's *t*-test. Probability values less than 0.05 (*p* < 0.05) were considered as statistically significant. Statistical analysis was performed using the GraphPad Prism 6.0 (GraphPad, San Diego, CA, USA) and Statistica 12.5 software (SigmaPlot, CA, USA).

## 3. Results

### 3.1. Ketamine Intermittent Administration in Adolescent Female Rats Does Not Alter Ambulation in the OF but Promotes Anxiogenic-like Behavior in the EPM Test

**Figure 1:**
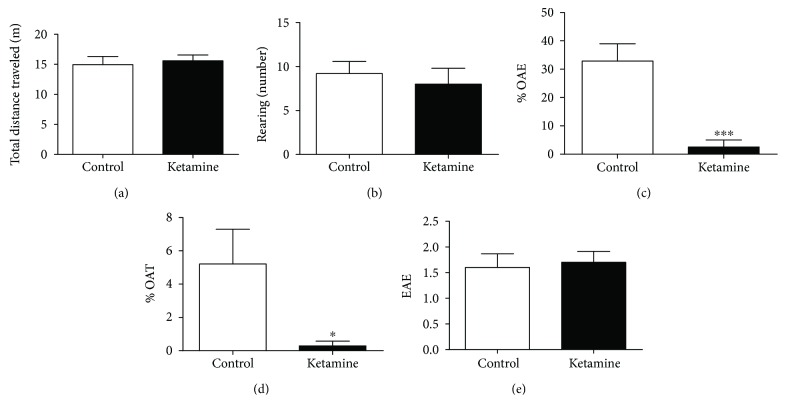
Effects of ketamine on the immediate withdrawal in adolescent female rats of 35th day until 37th day of life in the behavioral parameters: (a) traveled distance (meters) and (b) rearing number in the open-field (OF) test; (c) open arm entries (control percentage), (d) open arm time (control percentage), and (e) frequency of enclosed arm entries (number) in the elevated plus-maze. The results are expressed as the mean ± SEM (*n* = 10 animals per group). ^∗^*p* < 0.05 compared to the control group; ^∗∗∗^*p* < 0.001 compared to the control group (Student's *t*-test).

Administration of ketamine in the dose of 10 mg/kg for 3 consecutive days in female rat adolescents does not interfere in the animal ambulation (distance total traveled (*p* = 0.6977) and rearing number (*p* = 0.6011)) in the OF test (Figures 1(a) and 1(b)).

In the EPM test, ketamine decreased the % OAE (*p* = 0.0002; Figure 1(c)), as well as the % OAT parameter (*p* = 0.0313; Figure 1(d)), which suggests an anxiogenic-like effect. The EAE parameter was not altered in the animals treated with ketamine (*p* = 0.7730) (Figure 1(e)), which shows that the animals did not present motor behavior impairment, which corroborates with the findings in the OF test.

### 3.2. Ketamine Intermittent Administration in Adolescent Female Rats Promotes Depressive-like Behavior in the Forced Swimming Test

**Figure 2:**
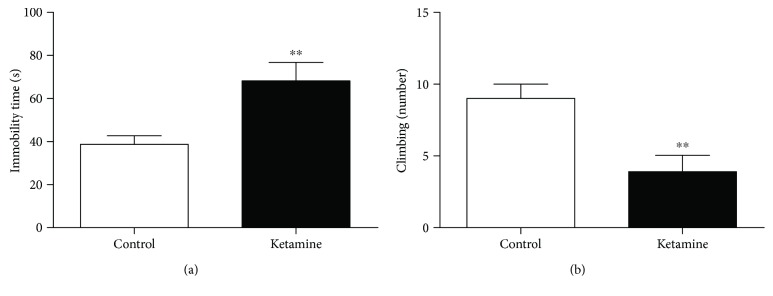
Effects of ketamine on the immediate withdrawal in adolescent female rats of 35th day until 37th day of life in the behavioral parameters: (a) immobility time (latency) and (b) climbing number. The results are expressed as the mean ± SEM (*n* = 10 animals per group). ^∗∗^*p* < 0.01 compared to the control group (Student's *t*-test).

The administration intermittent of ketamine for a period of 3 consecutive days led to a significant increase in the immobility time (*p* = 0.0059), 3 h after last injection. Moreover, ketamine intoxication showed reduction in the climbing number (*p* = 0.0037), which suggests a depressive-like effect (Figures 2(a) and 2(b)).

### 3.3. Ketamine Intermittent Administration in Adolescent Female Rats Induces Cognitive Impairment

**Figure 3:**
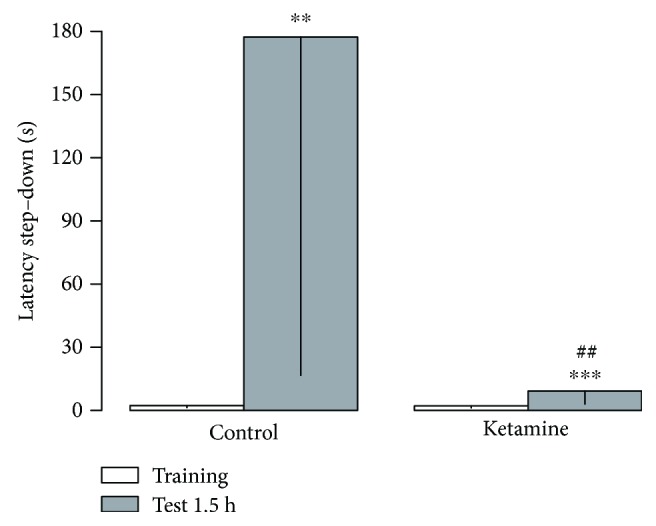
Effects of ketamine on the immediate withdrawal in adolescent female rats of 35th day until 37th day of life on short-term memory (1.5 hr). The results are expressed as the mean ± SEM. Data are shown as median (interquartile ranges) of latencies to step-down in the training and test sessions (*n* = 10 animals per group). ^∗∗^*p* < 0.01 compared to the training session of the same group; ^∗∗∗^*p* < 0.001 compared to the training session of the same group; ^##^*p* < 0.01 compared to the test stage of the control-treated group (Mann-Whitney).

The behavioral effects of ketamine on the immediate withdrawal in adolescent on the short-term memory are illustrated in Figure 3. Wilcoxon's test indicated that 3 h after the last administration of ketamine (at the dose of 10 mg/kg/day), animals reduced the step-down latency from the secure platform in the test session (*p* = 0.0017). Such result suggests that the ketamine impair short-term memory in adolescent rats (Figure 3).

### 3.4. Ketamine Intermittent Administration in Adolescent Female Rats Increases the Nitrites and Lipid Peroxidation Levels in the Blood and Hippocampus

**Figure 4:**
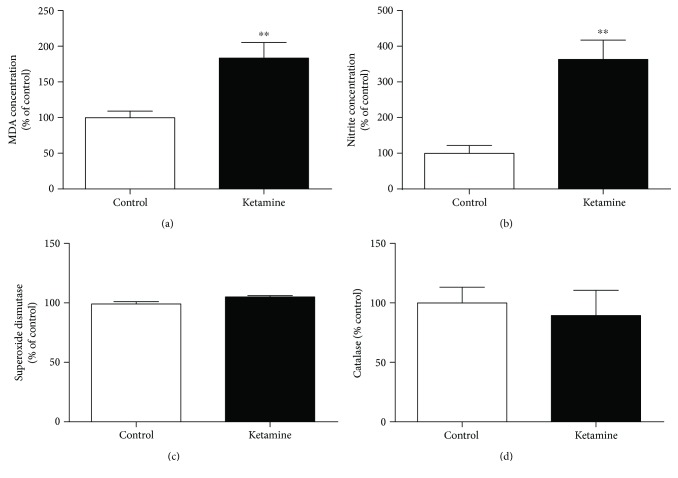
Effects of ketamine on the immediate withdrawal in adolescent female rats of 35th day until 37th day of life on oxidative stress on the blood samples. (a) Lipid peroxidation (malondialdehyde (MDA) concentration); (b) nitrite concentration; (c) superoxide dismutase; (d) catalase. The results are expressed as the mean ± SEM (*n* = 5 animals per group). ^∗∗^*p* < 0.01 compared with the control group (Student's *t*-test).

Ketamine administration increased the nitrites (*p* = 0.0041) and lipid peroxidation (*p* = 0.0063) levels in the blood samples (Figures 4(a) and 4(b)). However, the intermittent use of the drug did not change the serum levels of the antioxidants SOD (*p* = 0.0556) and CAT (*p* = 0.0661) enzymes (Figures 4(c) and 4(d)).

**Figure 5:**
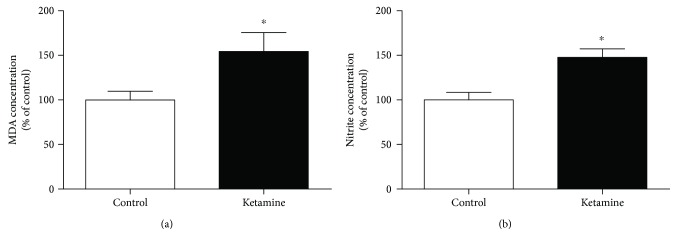
Effects of ketamine on the immediate withdrawal in adolescent female rats of 35th day until 37th day of life on oxidative stress on the hippocampus. (a) Lipid peroxidation (malondialdehyde (MDA) concentration); (b) nitrite concentration. The results are expressed as mean ± SEM (*n* = 5 animals per group). ^∗^*p* < 0.05 compared with the control group (Student's *t*-test).

In the hippocampus, the oxidative stress effects of ketamine on the immediate withdrawal in adolescent rats also increased the MDA (*p* = 0.0350; Figure 5(a)) and nitrite levels (*p* = 0.0110; Figure 5(b)), which suggest cell death and oxidative unbalance.

## 4. Discussion

This study demonstrates, for the first time, that ketamine intermittent administration in female rats on the adolescence period induced anxiety- and depression-like responses, as well as memory impairment, accompanied by hippocampal and systemic oxidative damage.

Ketamine essentially blocks NMDA receptors [45]. Glutamate is an essential neurotransmitter for the brain development and aging, as well as in the learning and memory process [46]. However, the blockade of glutamatergic receptors by ketamine promotes the accumulation of glutamate in the synaptic cleft [47], consequently, hyperstimulation of the glutamatergic pathway, which is extremely detrimental to the brain [48, 49].

Studies demonstrate that in the developing brain, especially in the period of early adolescent (prepubescent animals, PND 21-to-34) and midadolescent (periadolescent, PND 34–46) [50], NMDA-receptor antagonists have direct neurotoxic effects, affecting synaptogenesis or the brain growth during spurt period [51, 52]. Furthermore, excitotoxic cell death by excessive release of glutamate overestimates NMDA excitatory receptors to promote a different mechanism of apoptosis [53]. Besides, ketamine displays cell death in the developing brain by a mechanism involving a compensatory upregulation of NMDA receptor subunits, which in turn results in toxic accumulation of intracellular calcium, increased oxidative stress, and activation of the inflammatory pathways, becoming neurons more vulnerable [51, 52, 54].

Actually, the brain is especially sensitive to oxidative damage due to its extensive ability to consume large amounts of oxygen and the production of free radicals. In view of this, the process of oxidative stress is directly related to the onset of various brain diseases, including neurodegenerative disorders [55, 56] and psychiatric disorders [56, 57].

In the present study, the effects of ketamine in the immediate withdrawal promoted the increase in the MDA and NO_2_- systemic levels, without altering the antioxidant factors as the enzyme catalase and SOD. Excessive reactive species can cause oxidation of lipids releasing MDA; this is the final product of lipid peroxidation of polyunsaturated fatty acids. These lipids are susceptible to oxidative attack typically by ROS, thus, MDA is a biomarker for this type of damage and indicative of excessive release EROs [51]. The enzymatic antioxidant defense systems that suppress such ROS is understood as hydroxyl radicals (OH), superoxide anions (O_2_-), hydrogen peroxide (H_2_O_2_), superoxide dismutase (SOD), glutathione peroxidase (GPX), catalase (CAT), and thioredoxin peroxiredoxin (TRX-Prdx). These antioxidant enzymes can serve as redox biomarkers in different human diseases, because these antioxidant enzymes indicate the start of the redox state through oxidation/reduction processes. Although for SOD and CAT to be important antioxidants, it is not possible to observe difference in these antioxidants [58].

Increases of MDA and NO changes in the hippocampus demonstrate that this pathway is directly affected by intermittent ketamine administration, generating cell damage. According Patki et al. [59], the hippocampus seems to represent a common brain area that potentially mediates anxiety/depression-like behaviors and cognitive dysfunction induced by social defeat, in this way, drugs that promote hippocampal damage induce anxiety, depression, and memory deficits.

Anxiety is an aversive emotional state, in which the feeling of fear is disproportionate to the nature of the threat [56]. Against threatening situations, the feeling of the emotion constitutes subjective feature of anxiety that is accompanied by emotional stress and involves behavioral, expressive and physiological features, avoidance of the source of the danger, and assuming defensive postures [56, 60].

According Kuloglu et al. [61], there is a recently established link between certain anxiety behavior and oxidative stress, demonstrating that other systems, such as oxidative metabolism, can affect the regulation of anxiety. Thus, oxidative balance in the brain or plasma is important in anxious behavior. It is a valid highlight that the hippocampus exercises a pivotal role in anxiety and emotional-motivated behaviors [62]; thus, damage at this region after drug exposure induced by increasing of free radicals leads to oxidative damage in tissue, corroborating with our data.

Beyond the anxiogenic-like effects, the ketamine showed depressant-like activity in the FS test. Animals intermittently exposed to ketamine and subsequently evaluated after a final dose, present a significant increase in immobility time, as well as reduction of escalation. Studies indicate that ROS induced neuronal damage and has an important role in the pathophysiology of depression. Depression is characterized by the activation of the inflammatory response system with increased production of procytokines. Proinflammatory cytokines and cytokine-induced ROS may increase lipid peroxidation [63]. Reduced volumetric area, density, and glial cell number have been observed in the PFC and the hippocampus of patients with depression [64]. One of the most plausible causes for these neuronal alterations is elevated oxidative stress due to increased production of free radicals [65]. In fact, the hippocampus presents a significant role on mnemonic (dorsal part) as well as emotional behavior (ventral sub-region) [66]. Thus, we hypothesize that the changes found in FS can be explained by the oxidative imbalance in the blood and hippocampus of adolescent rats.

In addition to emotional profile, short-term memory was evaluated by the step-down inhibitory avoidance task. Our results demonstrated that female rat ketamine intermittent administration on the adolescence promoted impairment in short-term inhibitory avoidance memory. The hippocampus plays a pivotal role in fear aversive memory consolidation and retrieval [67] and conditioned fear memory paradigms [68]. We believe that ketamine-induced hippocampal oxidative damage could explain, at least in part, the mnemonic impairments observed in this study.

Actually, our results demonstrated that hippocampal-dependent behavior as anxiety and depression was modified by ketamine misuse in the adolescence. However, the locomotor activity, three hours after the last intermittent administration of ketamine, was preserved. It is noteworthy that spontaneous locomotor activity (horizontal and vertical) is primarily dependent of basal ganglia, cerebellum, and cerebral motor cortex (for review see Takakusaki [69]), which were not evaluated here. Thus, we suggest that our ketamine paradigm damage did not affect the motor-related brain areas sufficiently, which reflects on spontaneous motor behavior, even in the present systemic oxidative stress state.

The importance of this study relies on the major neurobehavioral changes associated with oxidative damage following ketamine misuse in the immediate withdrawal during adolescence. In fact, ketamine withdrawal symptoms characterized by anxiety, shaking, sweating, palpitations, and craving seem to be key problems in frequent ketamine users and have been proposed by many case studies. However, evidence of ketamine withdrawal in rodents at recreational or subanesthetic doses as well as during adolescence is scarce.

## 5. Conclusions

Overall, our data suggest that the effects of ketamine in the immediate withdrawal in the adolescence period promotes systemic and hippocampal oxidative stress, accompanied by emotional behavior alterations, i.e., anxiogenic and depressive profile, as well as mnemonic impairment, i.e., short-term memory, in the immediate withdrawal period. The present study consists of preliminary researchers focused on ketamine-misuse harmful effects. Thus, other works are necessary to investigate in detail other possible mechanisms that may contribute to oxidative stress in the behavioral alterations observed. In addition, the harmful effects of ketamine misuse during adolescence on brain structures are already unknown.

## Data Availability

The quantitative and qualitative data used to support the findings of this study are included within the article.
